# Cholesterol-lowering effect of *Lactobacillus rhamnosus* BFE5264 and its influence on the gut microbiome and propionate level in a murine model

**DOI:** 10.1371/journal.pone.0203150

**Published:** 2018-08-28

**Authors:** Soyoung Park, Jihee Kang, Sanghaeng Choi, Haryung Park, Eunchong Hwang, Yoongu Kang, Aram Kim, Wilhelm Holzapfel, Yosep Ji

**Affiliations:** 1 Advanced Green Energy and Environment, Handong Global University, Pohang, Gyungbuk, Republic of Korea; 2 AtoGen Co. Ltd., Daejeon, Republic of Korea; 3 Life Science, Handong Global University, Pohang, Gyungbuk, Republic of Korea; University of Louisville School of Medicine, UNITED STATES

## Abstract

Thanks to recent scientific progress a relationship between the intestinal microbiota and metabolic diseases could be established. A deeper understanding of underlying mechanisms has opened ways towards new approaches for alleviating conditions associated with metabolic diseases. Dysbiosis appears to be a major underlying factor associated with metabolic syndrome and related adverse health conditions. A major focus has therefore shifted to controlling of the gut microbiota through administration of functional lactic acid bacteria (LAB). The scope for health promotion and/or support by probiotics such as LAB has thereby been widened beyond the improving of intestinal health, also to include anti-obesity, anti-diabetic and cholesterol-lowering effects. In this study we investigated the cholesterol-lowering and microbiota modulatory potential of a LAB strain, *Lactobacillus rhamnosus* BFE5264, isolated from Maasai fermented milk. A mouse model receiving a high-cholesterol diet served as model for evaluating its functionality. The administration of *L*. *rhamnosus* BFE5264 resulted in a significant reduction of the serum cholesterol level that was accompanied by changes in intestinal microbiota and the production of short chain fatty acid (SCFA) in comparison to the control group. This strain also beneficially influenced the regulation of cholesterol metabolism in the liver in a pattern similar to that resulting from statin treatment, a drug inhibiting cholesterol biosynthesis in the liver.

## Introduction

Defining and explaining beneficial features of probiotics have become major issues of importance to scientists, industry and regulating authorities alike. Diverse factors have driven this development and include not only commercial and competitive marketing considerations, but also the paramount need for a scientific understanding of the role and functions of the diverse gut microbial communities in health and disease. Probiotics have been shown to improve gut homeostasis by modulating gut microbiota, and thereby improving beneficial interactions with host cells and nutrients [1]. Destabilisation of the gut microbiota, a condition also termed dysbiosis, appears to be related to various host disorders. An increasing number of studies is dealing with the impact of probiotics on metabolic diseases and on underlying mechanisms. As a result, probiotics have been proposed as a beneficial modulator of disrupted intestinal microbiota, a condition defined as dysbiosis and shown to be closely linked with symptoms of several metabolic disorders [2–5]. Still, precise mechanisms basic to beneficial modulatory changes in intestinal microbiota and how they impact the well-being of the whole body during probiotic administration are not fully understood yet.

Short chain fatty acids (SCFAs) are presently being explored in various approaches to explain the relationship between gut microbiota and the host. SCFAs are produced in the colon through active fermentation of indigestible oligosaccharides and fibre by particular intestinal bacteria. These fatty acids not only serve as energy sources in the body but also influence the regulation of lipid and glucose metabolism, immune cell development and gene expression [6–10]. The three major SCFAs, acetate, propionate and butyrate produced in the intestine are considered to play a diverse role due to their dissimilarity with regard to receptors and their distribution in the body. Moreover, while acetate is produced by most intestinal bacteria, only specific distinct populations of colon bacteria generate propionate and butyrate; for production of the latter members of Clostridium cluster XIVa appear to be of primary importance [11]. Propionate is considered to be beneficial in the alleviation of obesity and other metabolic diseases due to its reducing effects on lipogenesis and cholesterol synthesis in the liver [12–14].

In this study, we used a mouse model to investigate the cholesterol-lowering effect of the putative probiotic *Lactobacillus rhamnosus* strain BFE5264, isolated from Maasai fermented milk [15, 16]. In previous studies we have demonstrated the cholesterol-lowering potential of BFE5264 by promoting cholesterol efflux in THP-1 cells and down-regulating Niemann-pick C1-like 1 (NPC1L1) expression in Caco-2 cells [17, 18]. In fact, variable mechanisms for the cholesterol-lowering effect of probiotics have been reported [19–22]. One of the suggested underlying mechanisms appears to involve the bile salt hydrolase (BSH) activity of some probiotic strains. By deconjugation of bile salts several BSM-positive LAB strains lower the rate of reabsorption of bile acid (BA) in the small intestine so that an increased amount of cholesterol is required as precursor for the synthesis of BA, thereby ultimately reducing the total cholesterol level in the body [20, 21]. For example, the administration of BSH-positive probiotic strains in an animal model resulted in modulation of gut microbiota and the intestinal environment towards further promoting BA excretion as well as upregulation of BA synthesis in the liver [22]. Thus, using an atherosclerosis mouse model, our main focus in this investigation has been on the regulation of cholesterol metabolism by administration of strain BFE5264 and looking into possible underlying mechanisms. We have compared the efficacy of BFE5264 against hypercholesterolemia with lovastatin, a statin drug used for reducing hyperlipidaemia, and also determined the modification of intestinal microbiota, SCFA production, and the regulation of cholesterol metabolism.

## Materials and methods

### Animal study and bacterial preparation

All procedures were conducted under the approval of the Committee on the Ethics of Animal Experiments of Handong Global University and followed the guidelines of the Korean Association for Laboratory Animals. All necessary measures for minimising suffering of animals were taken during the entire experimental period. 3-week old C57BL/6JTacN male mice were obtained from Saeron Bio Inc (Korea) and divided into five groups as described in S1 Table after a one-week adaptation period. Animals were housed together in a cage for each group (N = 7–8 per group) at 23±1 °C, 55±10% humidity; a 12-hour light/dark cycle was maintained after adaptation for a week. Respectively high or low cholesterol diets (RD12336 and RD12337, Research Diets) and sterile water were given to the mice *ad libitum*. *Lactobacillus rhamnosus* BFE5264, deposited in the KCTC (Korean Collection for Type Cultures; WDCM 597) under the number SD1221, and *L*. *rhamnosus* GG (ATCC 53103) were grown in MRS broth (Difco Laboratories INC., USA) and sub-cultured at least twice after unstocking. Bacteria were prepared once a week, suspended in sterile PBS at 1x10^10^ CFU/400 μL and preserved in 4° C with continuous monitoring of the viability. 200 μL of either bacterial suspension, sterile PBS, or 30 mpk of lovastatin (Lovalord Tab., Chong Kun Dang Pharmaceutical, Korea) were administered twice per day (B.I.D.) through oral gavage. After nine weeks the animals were sacrificed by cervical dislocation and organs such as the liver and intestine were collected and stored immediately at -80 °C. Serum samples obtained by cardiac puncture were also collected by centrifugation of plasma at 2000 rpm for 30 min.

### Blood analysis

The amounts of total cholesterol, high-density lipoprotein (HDL) and triglyceride (TG) were determined using an assay kit (Asan Pharm. Co., Korea), while low-density lipoprotein (LDL) was calculated by the Anandaraja method [23].

### Analysis of mRNA expression in organs

mRNA expressed in the liver and the intestine was prepared with TRIzol Reagent (Invitrogen, USA). 50 mg of each samples were homogenised in 1mL Trizol using a T10 basic ULTRA-TURRAX (IKA, Germany) and centrifuged at 12000 g for 10 min. After adding 0.2 mL of chloroform and centrifuging 12000 g for 15 min, only the aqueous phase containing the RNA was transferred to a new tube; RNA precipitation was performed by addition of 0.5 mL of isopropanol. The RNA pellet formed after centrifugation at 7500 g for 10 min was washed with 70% ethanol and resuspended in RNase-free water after removal of the ethanol. The purity and concentration of RNA were measured by SPECTROstar (BMG LABTECH, Germany). 3 μg of cDNA (complementary DNA) were prepared using the GoScript^™^ Reverse Transcription System (Promega, USA) and a Verity 96-well thermal cycler (ABI Research, USA). Quantitative real-time PCR was performed by SYBR Premix Ex Taq^™^ II (Takara, Japan) with 20 ng of cDNA for each reaction using Step-One Plus real-time PCR System (Applied Biosystems, USA). Data were normalised by glyceraldehyde 3-phosphate dehydrogenase (GAPDH) expression of each organ. Specific primers used in this study are:

- *gapdh*_fw = 5’TGTGTCCGTCGTGGATCTGA,
- *gapdh*_rv = 5’CCTGCTTCACCACCTTCTTGA,
- *hmgcr*_fw = 5’CTTGTGGAATGCCTTGTGATTG, *hmgcr* rv = 5’
- AGCCGAAGCAGCACATGAT, *ldlr*_fw = 5’GAACTCAGGGCCTCTGTCTG,
- *ldlr*_rv = 5’ GAAACCATGCGTGTATCCCT,
- *cyp7a1*_fw = 5’AGCAACTAAACAACCTGCCAGTACTA, and
- *yp7a1*_rv = 5’GTCCGGATATTCAAGGATGCA.

### Microbiota analysis

Total DNA from caecal samples was extracted with QIAamp Stool Mini kit (Qiagen, USA) mainly according to the manufacturer’s instructions with an additional bead-beating step of 3 min using a Mini-beadbeater-16 and zirconia/silica 0.1 mm (Biospec, USA). All extracted DNA samples were sequenced using the Illumina MiSeq system of Chunlab (Seoul, Korea) and the V3-V4 variable regions of 16S rRNA amplicons (Forward Primer = 5' TCGTCGGCAGCGTCAGATGTGTATAAGAGACAGCCTACGGGNGGCWGCAG, Reverse Primer = 5' GTCTCGTGGGCTCGGAGATGTGTATAAGAGACAGGACTACHVGGGTATCTAATCC). Raw data generated from the sequencing was analysed using Qiime 1.9.1. A total of 2,901,140 raw reads were produced, comprising an average of 82,889 reads per sample. The raw reads were filtered out when the Phred quality score (Q score) was under 19. Chimeras were then eliminated using Vsearch 2.4.3. These two steps were processed by split libraries fastq.py and identifying_chimeric_seqs.py respectively from Qiime. 83.2% (2,412,667) of the reads were selected for clustering into operational taxonomic units (OTU), identified by open-reference picking using the UCLUST algorithm and taxonomy identification was assigned using the Greengenes database v13.8. These OTU picking steps were performed by pick_open_reference_otus.py from Qiime. Composition, alpha and beta diversities of taxonomically assigned microbiota were examined by core_diversity_analyses.py from Qiime. To generate unweighted UniFrac distance matrices, all communities were rarefied to 48,114 V3-V4 16S rDNA reads per sample. Plots of UniFrac distances are generated from unweighted analyses and UniFrac distance values are reported as within group versus between groups by using principal coordinates analysis (PCoA). The sequenced results have been deposited at the NCBI Sequence Read Archive database under the BioProject number PRJNA412298 (SRP119018).

### SCFA analysis

The method of Schwiertz et al (2009) was used to analyse SCFA in caecal contents. Volatile SCFAs were analysed in caecal samples by incubating 30 mg of specimen in 300 μL of buffer including 0.1 mole oxalic acid L^-1^ and 40 mmole sodium azide L^-1^ for one hour at room temperature, followed by centrifugation at 16,000 g for 10 min. Serum samples were directly analysed without any extraction procedure. The extracted SCFAs in the supernatant were analysed with Shimadzu GC2010 (Shimadzu, Japan) using a flame-ionized detector (FID). An HP INNO-WAS column (30 m x 32 mm) was used for analysis; its temperature was increased from 100 °C to 180 °C at 25 °C m^-1^ velocity with 27.1 psi pressure. The temperature of the splitter and FID was 260 °C. The concentration of each SCFA was calculated with a standard curve of peak retention time and concentration, derived from a volatile fatty acid mixture (Supelco, USA) as an external standard.

### Statistical analysis

All data are shown as mean and standard deviation. To compare the mean value among groups, one-way ANOVA was used with Fisher’s LSD *post hoc* analysis after Levene’s test of variance homogeneity by IBM SPSS Statistics 23 (IBM, USA). Significance was accepted at P<0.05.

## Results

### Cholesterol-lowering effect in a mouse model

**Fig 1:**
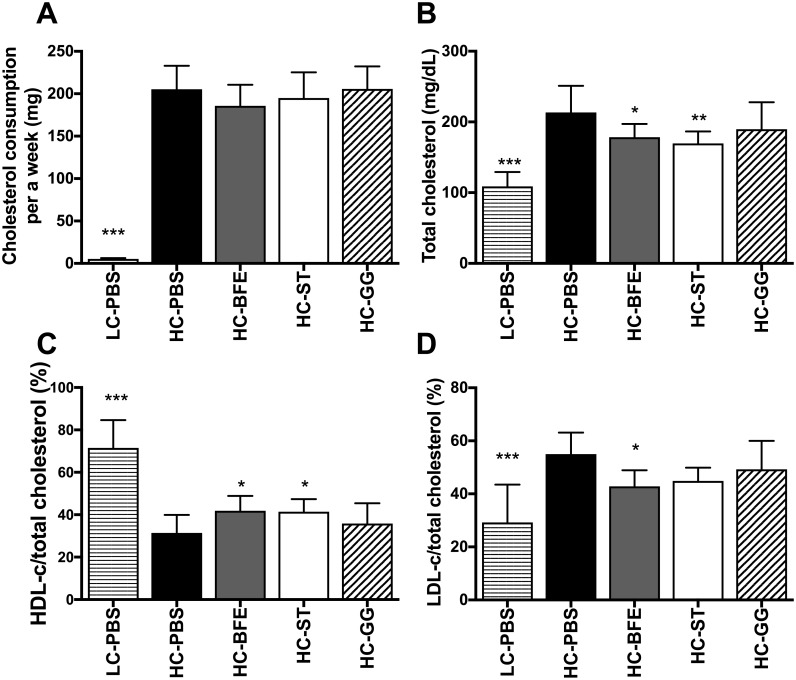
Cholesterol consumption of each mouse per week (A), and total amount of cholesterol (B) and percentage of HDL cholesterol (C) and LDL cholesterol (D) over total cholesterol in the serum. LC-PBS: low cholesterol control diet with sterile PBS; HC-PBS: high cholesterol control diet with sterile PBS; HC-BFE: high cholesterol diet group receiving *L*. *rhamnosus* BFE5264; HC-ST: high cholesterol diet group receiving lovastatin; HC-GG: high cholesterol diet group receiving *L*. *rhamnosus* GG. Data are shown as mean ± standard deviation and were analysed with one-way ANOVA. Significant differences compared to HC-PBS are presented as *asterisks*. *p<0.05; ** *p* <0.01; *** *p* <0.001.

To investigate the cholesterol-lowering effect of *L*. *rhamnosus* strain BFE 5264 in a murine model, a specific feed mixture for atherosclerosis research, with 1.25% of cholesterol and 0.5% of cholic acid (D12336, Research Diet, USA), was administered either together with each bacterial strain or with lovastatin as positive control. The concentrations of total serum cholesterol and low-density lipoprotein cholesterol (LDL-c) were significantly reduced, both in the group receiving strain BFE 5264 (HC-BFE) and the statin-treated group (HC-ST), whereas the beneficial high-density lipoprotein cholesterol (HDL-c) was increased in comparison to the control group receiving the high-cholesterol diet (HC-PBS) (Fig 1 and Table 1). Total weight gain of the HC-BFE and HC-ST groups was also less than that of HC-PBS (Table 1). Contrary to expectation, the triglyceride (TC) level of the low cholesterol control group (LC-PBS) strongly differed from other groups; this might be due to the different time points at which serum samples were collected in this group (Table 1). Still, experiments with other high-cholesterol consuming groups were performed under identical conditions as far as possible.

**Table 1:** Overall summary of animal experiment. LC-PBS: low cholesterol consuming control treated with sterile PBS; HC-PBS: high cholesterol consuming control treated with sterile PBS; HC-BFE: high cholesterol consuming group receiving *L*. *rhamnosus* BFE5264; HC-ST: high cholesterol diet group receiving lovastatin; HC-GG: high cholesterol consuming group receiving *L*. *rhamnosus* GG. HDL: high-density lipoprotein; LDL: low-density lipoprotein. Data are shown as mean ± standard deviation and were analysed with one-way ANOVA. Significant differences are presented by different letters.

	LC-PBS	HC-PBS	HC-BFE	HC-ST	HC-GG
Initial BW (g)	20.85±1.05	20.26±1.06	20.30±0.76	20.23±0.60	19.5±1.28
Final BW (g)	26.69±2.37	26.33±1.93	24.27±2.33	24.86±1.48	26.77±2.93
BW gain (g)	5.84±1.35^a^	6.06±1.10^a^	4.17±1.82^b^	4.64±0.97^ab^	7.03±2.02^a^
Liver (g)	1.12±0.11^a^	1.76±0.30^b^	1.69±0.24^b^	1.67±0.17^b^	1.68±0.17^b^
AT (g)	0.37±0.07	0.33±0.08	0.30±0.08	0.37±0.11	0.32±0.08
FC *	65.83±10.89	67.34±9.06	60.96±8.14	60.93±9.99	67.47±8.71
FE **	1.18±0.38	1.26±0.31	0.91±0.21	0.95±0.42	1.22±0.45
TC (mg/dL)	108.8±20.5^a^	213.4±37.8^b^	178.4±18.8^c^	169.6±16.9^c^	189.8±38.1^bc^
HDL (mg/dL)	76.4±11.7^a^	61.1±16.9^b^	74.8±14.3^a^	69.7±9.4^ab^	65.6±7.7^ab^
LDL (mg/dL)	33.9±21.5^a^	119.1±35.9^b^	76.5±14.5^c^	76.2±13.3^c^	96.3±36.2^bc^
TG (mg/dL)	200.5±59.3^a^	146.6±35.4^b^	144.3±21.7^b^	133.1±24.6^b^	124.4±28.0^b^

BW: body weight; AT: adipose tissue, FC: feed consumption; FE: feed efficiency ratio; TC: total cholesterol; HDL: high density lipoprotein; LDL: low density lipoprotein; TG: triglyceride;

*: kcal consumed per week for each mouse;

**: weight gain per total feed consumed (% of g/kcal)

### Cholesterol efflux and absorption in the intestine

Previous *in-vitro* studies have showed a cholesterol lowering potential of BFE5264 in THP-1 and Caco-2 cell lines by an increasing of cholesterol efflux concomitantly with a decreasing absorption [17, 18]. In this investigation, the cholesterol concentration in the mouse faeces was analysed (S1 Fig). Animals in all high-cholesterol-fed groups consumed similar amounts of cholesterol. Therefore, if BFE5264 administration resulted in the regulation of either cholesterol efflux and/or absorption, it could be assumed that faecal cholesterol may be changed. However, no significant differences in the faecal cholesterol levels of the groups could be detected. Moreover, mRNA expression of intestinal ATP-binding cassette transporters and NPC1L1 associated cholesterol efflux and absorption in the gut were also not changed in the HC-BFE group compared to the control (HC-PBS).

### Gut microbiota modulation

In order to obtain additional information on underlying mechanisms of the cholesterol-lowering function of BFE5264, gut microbiota in the caecal contents was analysed by the Illumine Miseq system. It was postulated that probiotic intake could stabilise the intestinal microbiota by beneficial modulation of a dysbiotic-associated population resulting from a high-fat diet.

**Fig 2:**
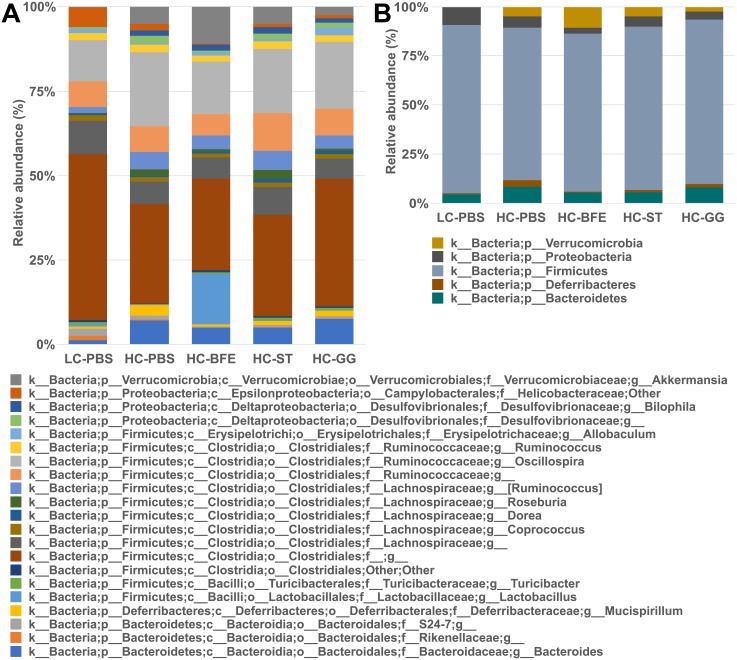
Relative abundance of assigned OTUs on genus level (A) and phylum level (B). The OTU picking was done by uclust taxonomy classifier by processing pick_open_reference_otus.py through Macqiime 1.9.1. LC-PBS: low cholesterol control diet with sterile PBS; HC-PBS: high cholesterol control diet treated with sterile PBS; HC-BFE: high cholesterol diet group receiving *L*. *rhamnosus* BFE5264; HC-ST: high cholesterol diet group receiving lovastatin; HC-GG: high cholesterol diet group receiving *L*. *rhamnosus* GG. Data are shown as mean value without standard deviation and OTUs with less than 0.05% of abundance were excluded.

Both BFE5264 and statin administration resulted in the modulation of *Bacteroidetes* and *Deferribacteres* populations to a level comparable with those of the LC-PBS (normal diet) group (Fig 2B). Especially the abundance of the phylum *Deferribacteres*, and more specifically the genus *Mucispirillum*, was significantly lower in these two feeding groups, equalling LC-PBS as compared to abundances in the control group (HC-PBS). LGG also induced a decrease in abundance although the change did not significantly differ from that of the control group (Fig 2A and 2B).

No overall change in the *Firmicutes* could be detected. This phylum accounts for the largest proportion of microbiota in the murine and human intestinal track (Fig 2B). It comprises numerous families and genera, and, when differentiating within this group, diet and probiotic feeding showed a significant impact on particular taxa. The abundance of the genus *Oscillospira* (family *Ruminococcaceae*) was higher in the HC-PBS group than in other groups, especially the LC-PBS and HC-BFE groups (P<0.003). In the family *Lachnospiraceae* the abundance of the genus *Roseburia* was decreased after probiotic (lactobacilli) but not of statin administration, while the abundance of the genus *Dorea* was affected by statin treatment. Interestingly, the genus *Lactobacillus*, which was significantly decreased after the administration of the high cholesterol diet, was not increased in the LGG group but only with BFE5264 administration (Fig 2A).

While the microbial population of the LC-PBS group contained a higher amount of the *Proteobacteria*, this phylum was mainly represented by the family *Helicobacteraceae*. On the other hand, the genus *Bilophila* (*Desulfovibrionaceae*) was detected in the high cholesterol-consuming groups but not in the LC-PBS group. BFE5264 administration significantly lowered the abundance of the family *Helicobacteraceae* and the genus-undefined family *Desulfovibrionaceae* compared to the HC-PBS group (P values 0.004 and 0.041, respectively) (Fig 2A and 2B).

A clear increase in the abundance of the phylum *Verrucomicrobia*, represented mainly by the genus *Akkermansia*, could be detected in the BFE5264-treated group, although with no statistical significance due to the huge variation between individuals (Fig 2).

**Fig 3:**
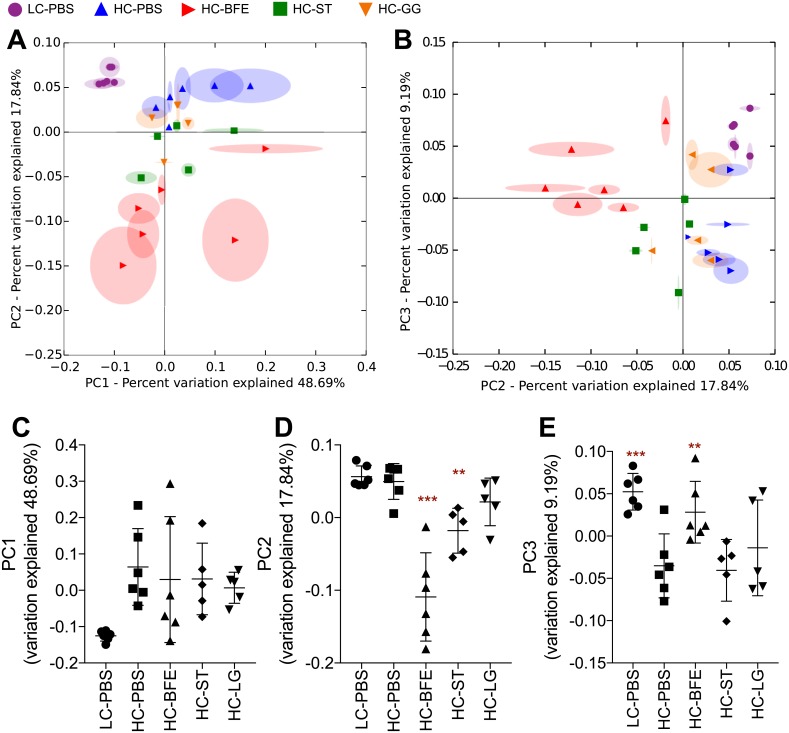
Principal coordinates analysis (PCoA) plots (A-B) and the score of each PC1 (C), PC2 (D) and PC (E) generated from weighted UniFrac distance metrics. LC-PBS: low cholesterol control diet with sterile PBS; HC-PBS: high cholesterol control diet with sterile PBS; HC-BFE: high cholesterol diet group receiving *L*. *rhamnosus* BFE5264; HC-ST: high cholesterol diet group receiving lovastatin; HC-GG: high cholesterol diet group receiving *L*. *rhamnosus* GG. Data are shown as mean ± standard deviation and were analysed with one-way ANOVA. Significant differences compared to HC-PBS are presented as *asterisks*. *p<0.05; ** *p* <0.01; *** *p* <0.001.

Beta diversity of the gut microbiota was by calculation of weighted unifrac distance. In the PCoA plots, each gut microbial population in the HC-BFE group was distinguished more strongly from the HC-PBS than either of the HC-ST or HC-GG groups (Fig 3A and 3B). LC-PBS was expectedly different from other groups even in PC1 containing the major part of the whole community (Fig 3C). BFE and statin treatment have shown significant differences compared to the control group along the PC2 axis, which seems to be related to the weight of the individuals (Pearson correlation between PC2 and weight gain was 0.481, p = 0.011) (Fig 3D). PC3 was significantly correlated with liver weight and serum, TC negatively and serum HDLc positively (Pearson correlations were -0.57, -0.635 and 0.407, respectively, p<0.005). Both HC-BFE and LC-PBS showed significantly higher levels in PC3 compared to the control group (Fig 3E).

### Increase of SCFAs in the intestine and the serum

In order to understand the relation between consumption of the bacterial strains and reduced cholesterol levels, we first expected that up-regulation of the BSH activity of microbiota modulated by BFE 5264 administration would increase BA excretion, thereby resulting in the use of more circulating cholesterol for *de novo* BA biosynthesis [9]. However, we could not find any results supporting this hypothesis, neither in the BA amount in the faeces nor in the expression of cholesterol 7-alpha-mono-oxygenase or cytochrome P450 7A1 (CYP7A1), an enzyme for BA synthesis, in the liver (S2 Fig). Against this background our focus shifted to the metabolites of the microbiota in the intestinal tract. Since SCFAs are well known for their impact on physiological activity, including lipid and glucose metabolism, the amounts of acetate, propionate, and butyrate were determined.

**Fig 4:**
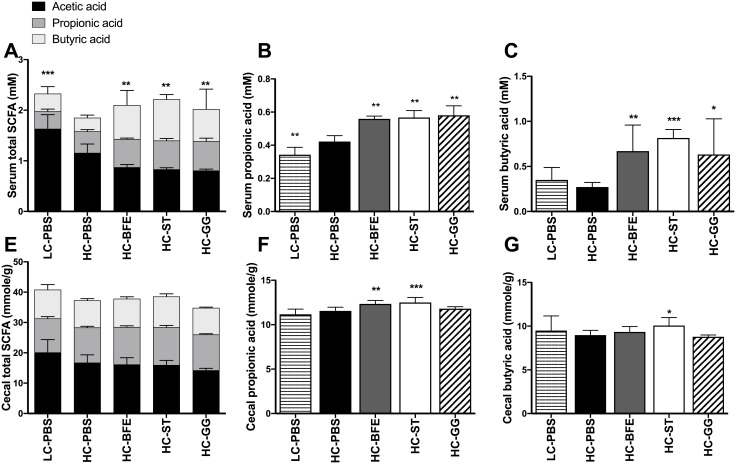
The amount of total short chain fatty acids (SCFA) (A), propionic acid (B) and butyric acid (C) in the serum, and caecal total SCFA (E), propionic acid (F) and butyric acid (G). LC-PBS: low cholesterol control diet with sterile PBS; HC-PBS: high cholesterol control diet with sterile PBS; HC-BFE: high cholesterol diet group receiving *L*. *rhamnosus* BFE5264; HC-ST: high cholesterol diet group receiving lovastatin; HC-GG: high cholesterol diet group receiving *L*. *rhamnosus* GG. Data are shown as mean ± standard deviation and were analysed with one-way ANOVA. Significant differences compared to HC-PBS are presented as *asterisks*. *p<0.05; ** *p* <0.01; *** *p* <0.001.

Compared to the low-cholesterol consuming group, high cholesterol consumption decreased the overall amount of the circulating SCFAs in the serum, and treatment with either the probiotic LAB or statin appears to support the recovering from the lack of circulating SCFAs. However, the composition of serum SCFAs showed patterns differing from those of the LC-PBS group, with lower acetate and higher propionate and butyrate concentrations (Fig 4A–4C). Even with a smaller modulation in caecal SCFAs and no significant change in the amount of total SCFAs among groups, the HC-BFE and HC-ST groups showed higher amounts of caecal propionate, compared to the HC-PBS group (Fig 4E and 4F). Again, no dramatic differences in faecal SCFAs were found among groups. Still, a slight decrease in faecal butyrate was detected in the treatment groups receiving the bacterial strains and statin, respectively, as compared to the controls (S3 Fig).

### Regulation of cholesterol biosynthesis in the liver

**Fig 5:**
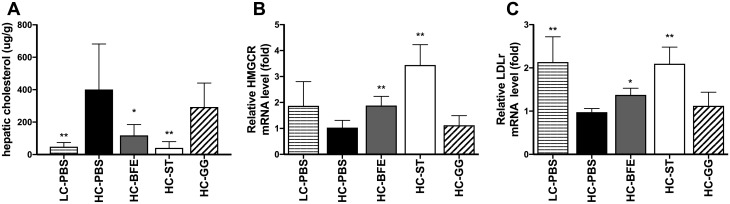
The amount of cholesterol in the liver (A) and the mRNA expression level of HMG-CoA reductase (B) and LDL receptor (C). LC-PBS: low cholesterol control diet with sterile PBS; HC-PBS: high cholesterol control diet with sterile PBS; HC-BFE: high cholesterol diet group receiving *L*. *rhamnosus* BFE5264; HC-ST: high cholesterol diet group receiving lovastatin; HC-GG: high cholesterol diet group receiving *L*. *rhamnosus* GG. Data are shown as mean ± standard deviation and were analysed with one-way ANOVA. Significant differences compared to HC-PBS are presented as *asterisks*. *p<0.05; ** *p* <0.01.

Concomitantly with the increase of propionate in the serum and caecum of the groups receiving strain BFE 5264 and statin, respectively, cholesterol levels were decreased, suggesting its possible influence on the modulation of hepatic cholesterol biosynthesis in this study. Cholesterol is synthesized by acetyl-coenzyme A activity in the liver involving 3-hydroxy-3-methylglutaryl-coenzyme A (HMG-CoA) reductase as a rate-limiting enzyme. Statin inhibits this HMGCR activity to lower cholesterol biosynthesis, resulting in the up-regulation of its mRNA expression (Fig 5B). As for statin, BFE 5264 also increased hepatic mRNA expression of HMG-CoA reductase and LDL receptor (Fig 5B and 5C). Furthermore, total cholesterol in the liver was significantly reduced, not only by statin but also by BFE 5264 (Fig 5A). On the other hand, administration of the probiotic LGG neither changed hepatic cholesterol levels, nor the mRNA expression of biomarkers associated with cholesterol metabolism in the liver (Fig 5).

## Discussion

People of the Maasai tribe in East Africa are known for their good health and the absence of symptoms related to metabolic syndrome. In spite of a high animal fat intake by the daily consumption of fermented milk as basic diet, low blood serum cholesterol levels of these tribal people have been reported as early as in the 1970’s [24]. Explanations for this so-called Maasai paradox have not been given conclusively, considering the high daily intake of unsaturated fats and cholesterol, typical of a diet causing alarm for populations with “Western” lifestyle. Based on an earlier “milk-factor” hypothesis, the interaction between several factors has been proposed as explanation for the cholesterol-lowering effect of milk [25]. We postulate a decisive role for (lactic acid) bacteria associated with the fermentation of Maasai milk in the regulation of cholesterol. *L*. *rhamnosus* BFE5264, isolated from Maasai fermented milk [15], was selected for this investigation. In former reports the safety and potential of this strain as a probiotic have been confirmed [26].

Initially, using a Caco-2 cell line model, we have shown that the decrease of cholesterol uptake was related to the inhibition of NPC1L1 and the up-regulation of ABCG5/6 through LXR activation by strain BFE5264 [17, 18]. In this study, we investigated the strain’s cholesterol-lowering effect in an animal model. Even when the down-regulation of NPC1L1 expression in the intestine or changes in the amount of cholesterol in faeces has not been determined in this *in vivo* study, the strain’s cholesterol-lowering effect could be confirmed in the murine model consuming a high-cholesterol feed. Total serum cholesterol, LDL cholesterol and hepatic cholesterol were reduced significantly while, compared to the high-cholesterol control group, the beneficial serum HDL cholesterol was increased after BFE5264 administration.

To comprehend the cholesterol-lowering effect of *L*. *rhamnosus* BFE5264 in an animal model, we focused on the changes of intestinal microbiota and their metabolites, and on the key biomarkers that could be determined both in the animal and cell line experiments. According to Sheng et al [26], the abundance of the families *Deferribacteraceae* and *Helicobacteraceae* in the caecal microbiota is positively correlated with the concentation of serum cholesterol. In our study, high cholesterol consumption significantly increased the abundance of the *Deferribacteaceae*, especially of the genus *Mucispirillum*, while BFE5264 and statin administration, respectively, “normalised” their abundance in the caecal microbiota, also when compared to the low cholesterol diet (LC-PBS). BFE5264 treatment also reduced the abundance of the families *Helicobacteraceae* and *Desulfovibrionaceae* compared to the high cholesterol control group (HC-PBS), resulting in a noticeable change in the overall numbers of the phylum *Proteobacteria* (Fig 2). On the other hand, the abundance of the genus *Lactobacillus*, known as the beneficial bacterial subset, was increased in the caecum following BFE 5264 administration. In fact, *Lactobacillus* spp. are considered to represent one of the major groups in the small intestine rather than in the caecum or colon because of their tolerance against low pH and bile acid (in the duodenum) and their oxygen tolerance [27]. However, in our study, the administration of BFE5264 increased the relative abundance of lactobacilli to 14.76 (± 9.97) % in the caecum. Further investigation of the effect of BFE5264 on the microbiome in the small intestine is expected to provide a broader understanding of the mode of efficacy of this strain and its impact on the *Lactobacillus* population. Interestingly, although LGG and BFE5264 belong to the same species (*L*. *rhamnosus*), the results reflected clear yet diverse modulatory differences in their impact on gut microbiota and the resulting cholesterol reduction. These results strongly underline the importance of strain-specificity when referring to measurable effects based on gut microbiota modulation and functional efficacy in an *in vivo* model.

Lovastatin, the positive control in this study, was originally isolated from *Monascus purpureus* and inhibits the activity of HMG-CoA reductase, a rate-limiting enzyme of cholesterol biosynthesis, to lower cholesterol synthesis and thus to treat hypercholesterolemia. Statin treatment impacting the amount of hepatic cholesterol increases the mRNA expression of HMG-CoA reductase. When BFE5264 was administrated to high-cholesterol consuming mice, mRNA expression of this enzyme was also increased as with statin treatment. In addition, expression of LDL receptor and LXR was also increased in both groups (BFE5264 and statin). Although the actual inhibition of the HMG-CoA reductase activity by BFE5264 was not determined in this study, hepatic cholesterol was significantly reduced compared to the control group after BFE5264 treatment. Also, expression of biomarkers associated with lipid metabolism in the liver was regulated by this strain, suggesting a role of BFE5264 towards maintaining cholesterol homeostasis in the liver even after high-cholesterol consumption. One possible explanation appears to be related to gut microbiota modulation following BFE5264 administration, resulting in qualitative and quantitative changes in metabolites such as SCFAs in the intestinal tract. Propionic acid, one of the three major SCFAs, is known for its role in lipid metabolism in the liver and several papers have reported its influence on cholesterol biosynthesis in the liver [13, 14, 28]. We have shown that both BFE5264 and statin treatment specifically increased the amount of propionic acid in the caecum. As a *Lactobacillus*, BFE5264 does not produce propionic acid, and thus the increase of propionic acid in the intestinal track can only be explained by its modulatory effect on the microbial population, resulting in an increased production of propionate and other SCFA producing strains. Finally, even though propionic acid was produced actively by the modulated microbiota, a relatively stable amount of cholesterol most probably seems to be maintained in the liver and serum regardless of the elevated intake of cholesterol. Moreover, the increase in circulating butyrate detected in both *Lactobacillus*-fed groups seems not to be correlated with or reflected in the (insignificant) changes in the caecum butyrate. This may be explained partly by further studies on the absorption and utilization of butyrate showing that the amounts of excreted (faecal) butyrate were significantly smaller in the respective groups receiving the lactobacilli and statin (S3D Fig). The role of butyrate in lipid metabolism and metabolic diseases has been mentioned in several reports. Yet, mechanisms underlying changes in the cholesterol level relative to butyrate (and propionate) production after *Lactobacillus* administration warrant further clarification [6–10].

This research emphasises a desirable improvement of the intestinal environment induced by probiotics as compared to the direct and sometimes dramatic effects resulting from the intake of statins and other synthetic drugs. We expect that systematic *in vivo* studies will lead to a deeper understanding and linking of interactions among the various organs and provide mechanistic insights in the beneficial effects of probiotics as compared to synthetic drugs that may frequently lead to undesired side-effects. There is general agreement on the safety and acceptability of proven probiotics, yet, information and understanding of underlying mechanisms is still sparse. The cholesterol-lowering activity of BFE5264 relative to its influence on the intestinal tract was shown in this study. Nonetheless, some of these data differed from those our previous *in-vitro* studies [16, 17], and may be explained in future studies.

Through this study we have demonstrated a dynamic way of explaining various beneficial yet strain-specific effects of two LAB strains of the same species, *L*. *rhamnosus* (LGG and BFE5264), in particular with regard to differences in their cholesterol lowering ability.

## Supporting information

S1 Fig The amount of cholesterol in faeces (A) and the mRNA expression of ABCG5 (B) ABCG8 (C) and NPC1L1 (D) in the intestine. LC-PBS: low cholesterol consuming control treated with sterile PBS; HC-PBS: high cholesterol consuming control treated with sterile PBS; HC-BFE: high cholesterol consuming group receiving *L*. *rhamnosus* BFE5264; HC-ST: high cholesterol diet group receiving lovastatin; HC-GG: high cholesterol consuming group receiving *L*. *rhamnosus* GG. Data are shown as mean ± standard deviation and were analysed with one-way ANOVA. Significant differences compared to HC-PBS are presented as asterisks. *p<0.05; *** *p* <0.001.
(EPS)

S2 Fig The amount of bile acids in the intestine (A) and faeces (A), and the mRNA expression of CYP7A1 in the liver (B). LC-PBS: low cholesterol consuming control treated with sterile PBS; HC-PBS: high cholesterol consuming control treated with sterile PBS; HC-BFE: high cholesterol consuming group receiving *L*. *rhamnosus* BFE5264; HC-ST: high cholesterol diet group receiving lovastatin; HC-GG: high cholesterol consuming group receiving *L*. *rhamnosus* GG. Data are shown as mean ± standard deviation and were analysed with one-way ANOVA. Significant differences compared to HC-PBS are presented as asterisks. *** *p* <0.001.
(EPS)

S3 Fig The overall amount of SCFA (A) and acetic acids (B), propionic acids (C) and butyric acid (D) in faeces. LC-PBS: low cholesterol consuming control treated with sterile PBS; HC-PBS: high cholesterol consuming control treated with sterile PBS; HC-BFE: high cholesterol consuming group receiving *L*. *rhamnosus* BFE5264; HC-ST: high cholesterol diet group receiving lovastatin; HC-GG: high cholesterol consuming group receiving *L*. *rhamnosus* GG. Data are shown as mean ± standard deviation and were analysed with one-way ANOVA. Significant differences compared to HC-PBS are presented as asterisks. *p<0.05; ** *p* <0.01; *** *p* <0.001.
(EPS)

S1 Table Design of the experimental grouping. Animals were housed together in a cage for each group (N = 7–8 per group) at 23±1 °C, 55±10% humidity, in a 12-hour light/dark cycle after adaptation for a week. The experiment was performed for nine weeks subsequent to a one-week adaptation on a normal chow diet.
(DOCX)
