# Correction: Cholesterol-lowering effect of *Lactobacillus rhamnosus* BFE5264 and its influence on the gut microbiome and propionate level in a murine model

**DOI:** 10.1371/journal.pone.0208294

**Published:** 2018-11-26

**Authors:** Soyoung Park, Jihee Kang, Sanghaeng Choi, Haryung Park, Eunchong Hwang, Youn-Goo Kang, Ah-Ram Kim, Wilhelm Holzapfel, Yosep Ji

The sixth author’s name is spelled incorrectly. The correct name is: Youn-Goo Kang.

The seventh author’s name is spelled incorrectly. The correct name is: Ah-Ram Kim.

## References

[1] Park S, Kang J, Choi S, Park H, Hwang E, Kang Y, et al (2018) Cholesterol-lowering effect of *Lactobacillus rhamnosus* BFE5264 and its influence on the gut microbiome and propionate level in a murine model. PLoS ONE 13(8): e0203150 10.1371/journal.pone.0203150 30153290 PMC6112659

