# Supplementary material for: Cholesterol-lowering effect of Lactobacillus rhamnosus BFE5264 and its influence on the gut microbiome and propionate level in a murine model
Source: PLoS One. 2018 Aug 28;13(8):e0203150. doi: 10.1371/journal.pone.0203150 (PMC6112659; doi:10.1371/journal.pone.0203150)
Supplement: S1 Table — Animals were housed together in a cage for each group (N = 7–8 per group) at 23±1 °C, 55±10% humidity, in a 12-hour light/dark cycle after adaptation for a week. The experiment was performed for nine weeks subsequent to a one-week adaptation on a normal chow diet. (DOCX) [file pone.0203150.s004.docx]

| **Groups** | **Number**  **of mice** | **Diet** | | **Oral gavage (twice a day)** |
| --- | --- | --- | --- | --- |
| LC-PBS | 8 | low-cholesterol diet (D12337, Research Diet, USA) | | sterile PBS |
| HC-PBS | 8 | high-cholesterol diet (D12336^a^, Research Diet, USA) | | sterile PBS |
| HC-BFE | 7 | high-cholesterol diet (D12336^a^, Research Diet, USA) | | 1x10^10^ CFU of *L. rhamnosus* BFE5264^b^ suspended in sterile PBS |
| HC-ST | 8 | high-cholesterol diet (D12336^a^, Research Diet, USA) | | 30mpk of lovastatin^c^ |
| HC-GG | 7 | high-cholesterol diet (D12336^a^, Research Diet, USA) | | 1x10^10^ CFU of *L. rhamnosus* GG^d^ suspended in sterile PBS |
| ^a^ 1.25% cholesterol and 0.5% Cholic acid | | |  | |
| ^b^ WDCM 597 (Korean Collection for Type Cultures) | | |  | |
| ^c^ Lovalord Tab. ChongKunDang Pharmaceutical, Korea | | |  | |
| ^d^ ATCC 53103 | | |  | |

**S1 Table. Design of the experimental grouping.** Animals were housed together in a cage for each group (N = 7-8 per group) at 23±1 °C, 55±10 % humidity, in a 12-hour light/dark cycle after adaptation for a week. The experiment was performed for nine weeks subsequent to a one-week adaptation on a normal chow diet.
